# Enhanced Endoscopic Internal Drainage of Gastric Abscess Through Additively Manufactured Stents

**DOI:** 10.1002/adhm.202505860

**Published:** 2026-04-02

**Authors:** Parima Phowarasoontorn, Yongbin Ko, Juan S. Barajas‐Gamboa, Juan P. Pantoja, Oraib Al‐Ketan, Mohamed Ali, Sungyun Sohn, Heba Tageldeen Naser, Abdel‐Hameed Dabbour, Batoul Khlaifat, Ahmed AlZubaidi, Carlos Abril Vega, John Rodriguez, Matthew Kroh, Khalil B. Ramadi

**Affiliations:** ^1^ Division of Engineering New York University Abu Dhabi Abu Dhabi UAE; ^2^ Center For Translational Medical Devices New York University Abu Dhabi Abu Dhabi UAE; ^3^ Center For Brain and Health New York University Abu Dhabi Abu Dhabi UAE; ^4^ Digestive Disease Institute Department of General Surgery Cleveland Clinic Abu Dhabi Abu Dhabi UAE; ^5^ Core Technology Platform New York University Abu Dhabi Abu Dhabi UAE; ^6^ Division of Science New York University Abu Dhabi Abu Dhabi UAE; ^7^ Digestive Disease and Surgery Institute Cleveland Clinic Cleveland Ohio USA; ^8^ Tandon School of Engineering New York University New York New York USA; ^9^ Grossman School of Medicine and NYU Langone Health New York University New York New York USA

**Keywords:** 3D printing, anastomotic leaks, bariatric surgery, computational fluid dynamics (CFD), stents

## Abstract

Postoperative gastric leaks (GL), a serious complication of bariatric surgery, are often managed by endoscopic internal drainage using biliary double‐pigtail stents (DPS). However, conventional extruded thermoplastic (e.g., polyethylene, polyurethane, and polytetrafluoroethylene) stents often provide inadequate drainage, suboptimal anatomical conformity, and are prone to migration, collectively hindering patient recovery and contributing to an adverse event rate of 13.8%. To address these limitations, we developed PETALS (Personalized Endoscopic Transmural Abscess Leak Solution), an analytical framework for the optimization of stent designs dedicated to transmural fluid drainage. We identified stent length and diameter as critical geometric parameters influencing fluid dynamics. Using PETALS, we designed a family of stents with longitudinal fins fabricated by stereolithography (SLA) using Formlabs Flexible 80A resin. The Lily design, a six‐segment PETALS construct, achieved a 32% reduction in hydraulic resistance and a 30% increase in flow rate compared with a commercial DPS in a benchtop GL model, while also exhibiting superior flexibility. PETALS thus enables the fabrication of patient‐specific, additively manufactured stents with increased flow rate, potentially reducing complications and shortening hospitalization.

## Graphical abstract

Postoperative gastric leaks are often treated with off‐label biliary double‐pigtail stents, yet conventional extruded designs are not optimized for leak anatomy, can migrate, and may limit abscess evacuation. PETALS is introduced to optimize transmural drainage geometry and enable patient‐specific 3D‐printable stents. A finned “Lily” stent fabricated by stereolithography reduces hydraulic resistance and increases flow in a benchtop leak model.

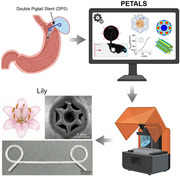

## 1 Introduction

**FIGURE 1:**
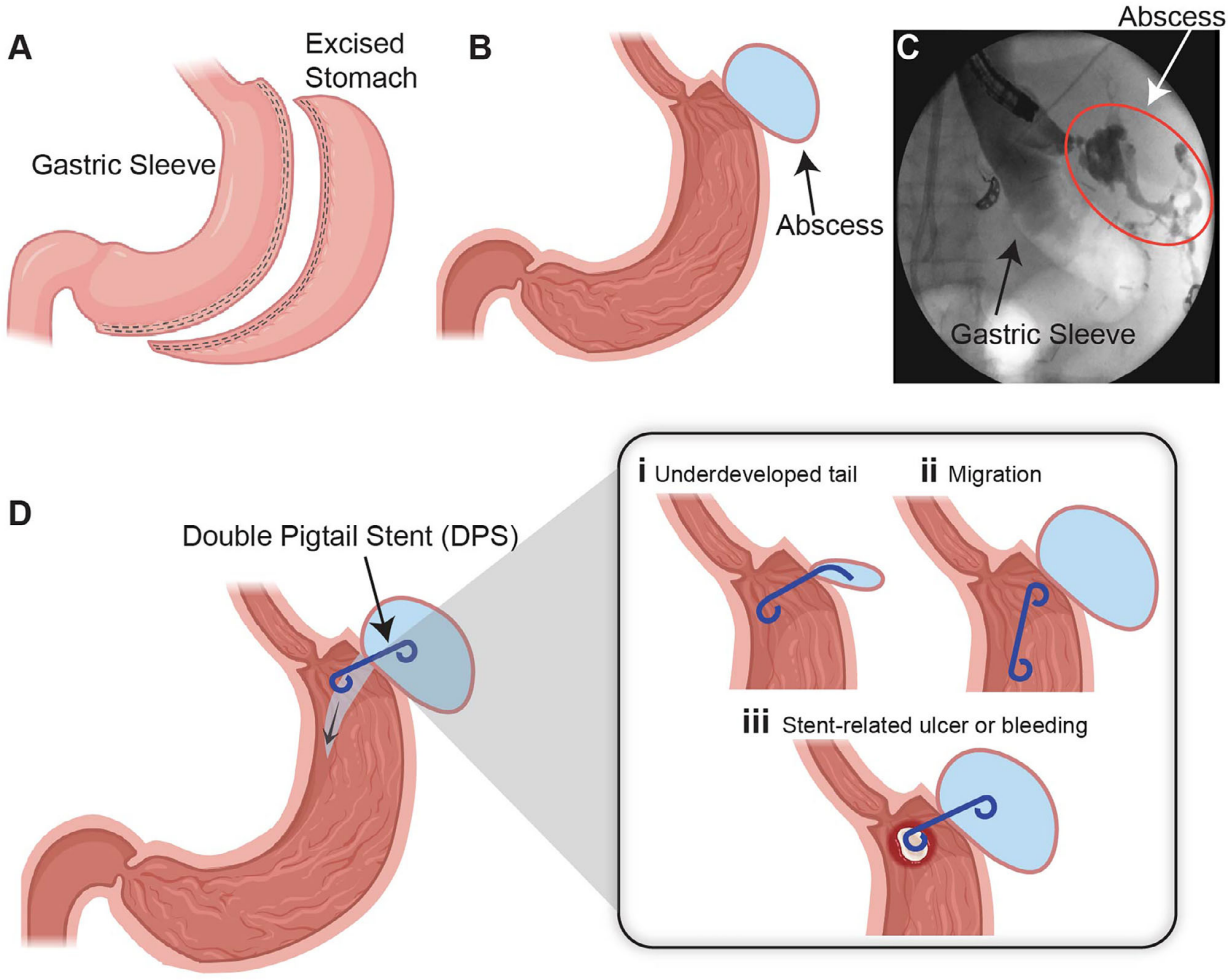
Overview of the surgical procedure. (A) Anatomical illustration of the sleeve gastrectomy surgical site. (B,C) post‐operative perigastric abscess formation. (D) Limitations of current off‐label biliary double pigtail stent (DPS) in gastric leak treatment application: (i) inadequate loop dimensions relative to abscess morphology can prevent full anchoring tail deployment; (ii, iii) suboptimal stent sizing leading to migration risks, mucosal ulceration, and bleeding.

Bariatric surgery is a widespread therapy for the management of morbid obesity and related cardiovascular diseases with proven long‐term results. Among the available procedures, laparoscopic sleeve gastrectomy (LSG) stands out as one of the most widely performed interventions due to its effectiveness and favorable safety profile (Figure 1A). With over 250,000 LSG procedures performed annually in the United States alone, even the relatively low complication rate translates to a substantial patient population affected by serious adverse events [1]. Despite significant advances in surgical techniques, complications such as gastric leaks (GL) remain a significant concern, occurring in 1–3% of primary cases and up to 10% in revision procedures, leading to prolonged hospitalizations and increased morbidity (Figure 1B,C) [2]. This represents approximately 2500 patients in the U.S. annually requiring GL management, with treatment costs ranging from $8000 to over $100 000 per patient depending on severity and duration [2, 3].

Current clinical standards for treating post‐surgical GL involve a range of techniques tailored to the severity and anatomical location of the leak [4]. Endoscopic techniques have become popular as less invasive alternatives to surgical re‐intervention [5, 6]. These techniques include self‐expanding metal stents (SEMS), through‐the‐scope clips (TTSC), over‐the‐scope clips (OTSC), tissue sealants, suturing systems, and endoscopic internal drainage (EID) utilizing double pigtail stents (DPS) [7, 8]. Notably, EID with DPS has become widely adopted by clinicians for GL management due to its high success rates of up to 89.5%, low adverse event rates of 13.8%, and high patient tolerance [5, 9, 10].

Despite these advantages, the current DPS options available on the market are primarily designed for biliary drainage, making their use in GL treatment off‐label [11]. Biliary stents are created for the anatomy of the bile ducts (typically 6–8 mm diameter, 5–15 cm length), whereas gastric leak cavities present highly variable geometries with abscess diameter ranging from 20–100 mm and irregular shapes that may not conform to circular structures of the stent's anchoring tails. This anatomical mismatch results in a lack of off‐the‐shelf options in various shapes and sizes, which restricts surgeons’ ability to select stents that best fit individual patient GL anatomy. Clinical evidence suggests that commercially available DPS sizes are often unsuitable for managing larger abscesses, as the stent tail may fail to hold in place, leading to dislodgement, inadequate drainage, and ultimately, treatment failure (Figure 1D) [12]. Additionally, stent migration remains a primary concern during EID procedures, with improper sizing serving as a major contributing factor. Migration can lead to life‐threatening complications, such as splenic injury and hemorrhage, often necessitating further surgical interventions [13].

Currently, DPS and other plastic stents are manufactured from polyethylene, polytetrafluoroethylene, or polyurethane using traditional processes such as extrusion and cutting [14]. Although these methods are cost‐effective, they lack the flexibility required for rapid prototyping and patient‐specific customization. To date, no indicated EID device has been specifically developed and optimized for gastric leak management, representing a significant unmet clinical need. Efficient GL cavity evacuation depends on establishing sufficient drainage under physiologically relevant pressure heads while minimizing flow resistance. Because hydraulic performance is strongly influenced by lumen cross‐sectional geometry and effective flow‐path length, we hypothesized that stents with non‐circular lumens could improve drainage relative to conventional circular designs without compromising mechanical properties. In parallel, additive manufacturing (AM) can enable design features that are difficult to realize with traditional manufacturing while also allowing rapid iteration and potential patient‐specific sizing to better match heterogeneous abscess anatomy.

Here, we report Personalized Endoscopic Transmural Abscess Leak Solution (PETALS), a family of stents developed through a combined analytical/empirical framework for optimized drainage. PETALS leverages the flexibility of AM technologies to create customizable patient‐specific DPS. Using computational fluid dynamics (CFD) simulations, we analyzed the working principles of existing DPS designs to identify key geometrical features for further optimization. We developed an analytical framework based on a modified Hagen‐Poiseuille equation to predict hydraulic resistance as a function of stent geometry, enabling rapid design iteration. Finally, we evaluated the fluid evacuation performance of our redesigned stent using a GL benchtop model, using a commercially available DPS as the baseline for comparison.

## 2 Results

### 2.1 Effects of Geometrical Parameters on Standard Stent Fluid Evacuation Performance

We first sought to understand the characteristics of fluid flow through a DPS. We designed a 2D CFD simulation to identify geometrical features of the stent and abscess that may affect the rate of fluid evacuation in pressure‐driven flow.

#### 2.1.1 Parametric Study of DPS Geometrical Features

**FIGURE 2:**
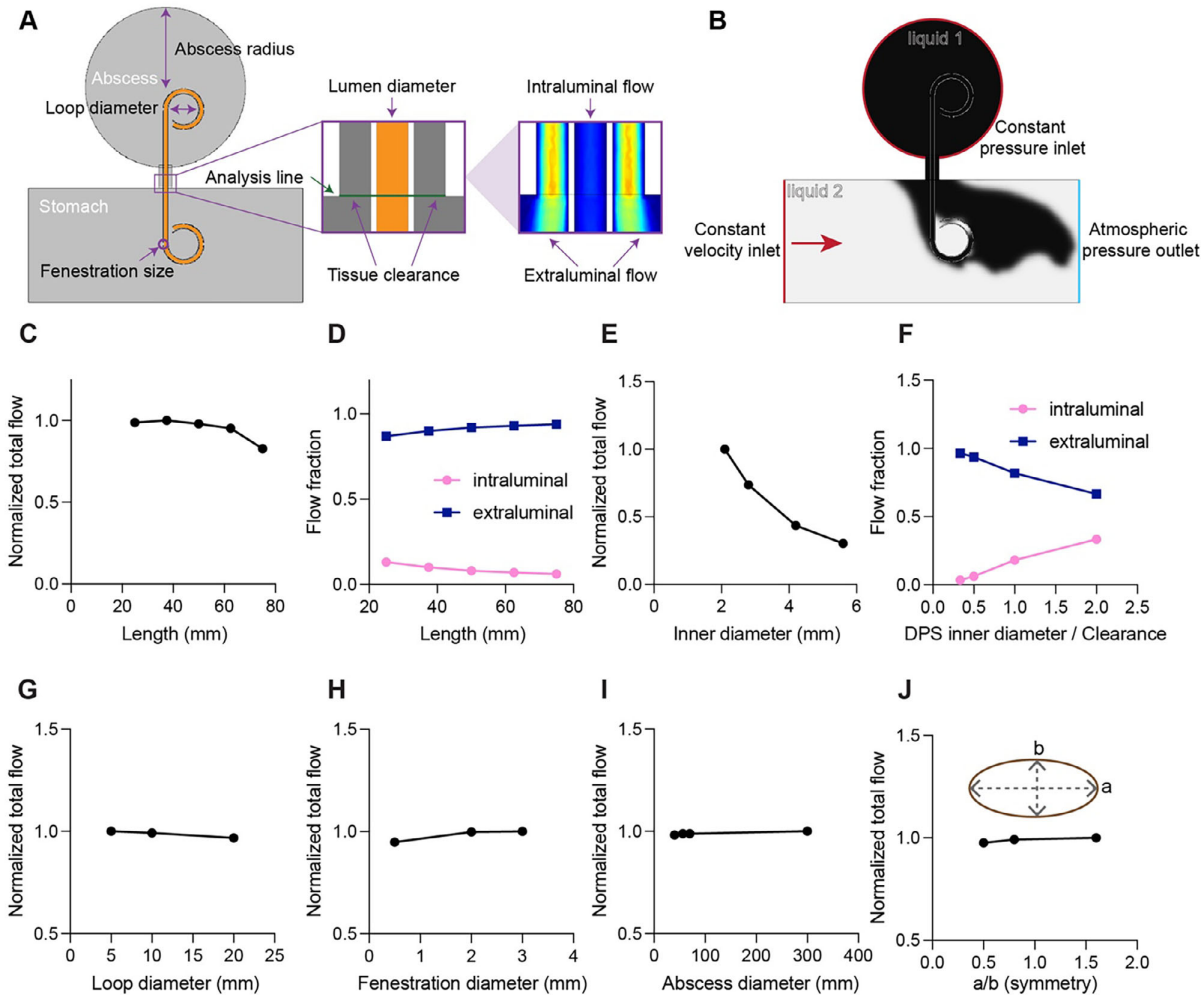
Computational fluid dynamics methodology. (A) 2D CFD simulation model with annotated geometrical parameters. (B) Boundary condition setup featuring pressure inlet (abscess cavity) and outlet (stomach terminal) with laminar flow, no‐slip conditions, and Newtonian fluid assumptions. Velocity profiles along the indicated green analysis line were sampled over 10 s duration. Results were then normalized using min‐max scaling relative to peak mass transfer values across each experimental condition. CFD parametric across dimensional variations: (C,D) DPS length (25–75 mm), (E‐F) lumen diameter (2.1–5.6 mm), (G) loop diameter (5–20 mm), (H) fenestration diameter (0.5–3.0 mm), (I) abscess diameter (40–300 mm), and (J) cavity eccentricity (aspect ratio 0.5–1.6).

We performed parametric studies to evaluate the effects of DPS length, internal diameter, tail loop diameter, and side‐hole diameter on the total liquid volume transferred over a 10‐s interval (Figure 2A,B). We applied a pressure‐driven flow boundary condition of 5 mmHg (668 Pa), chosen to reflect typical adult intra‐abdominal pressure [15]. We selected a characteristic inlet velocity of 0.04 ms^−1^ based on published measurements of fed‐state gastric mixing, which report intragastric flow velocities on the order of 3–7.6 cms^−1^ [16, 17]. Increasing the DPS length from 25 to 75 mm reduced total flow by 17.3% (Figure 2C). This length range encompasses the typical dimensions of gastric leak cavities observed in clinical practice, where the distance from the gastric lumen to the abscess cavity center varies with patient anatomy. Under a constant tissue clearance‐to‐inner diameter ratio of 2, external flow consistently dominated internal flow across all tested lengths (Figure 2D). Mechanistically, increasing DPS length extends the internal fluid pathway, raising its hydraulic resistance due to the direct proportionality between hydraulic resistance and path length under the Hagen‐Poiseuille framework. This elevated internal resistance preferentially diverts fluid to the external pathway, which offers lower resistance due to its shorter path length. Despite the shift, the combined resistance of both pathways exceeds that of the shorter DPS configuration, causing the total flow rate to decrease. Increasing the inner diameter from 2 mm to 6 mm, while keeping the overall tissue opening size constant, decreased total flow by 69.72% (Figure 2E,F). This counterintuitive finding suggests that increasing the inner diameter reduces fluid evacuation, as the resulting decrease in tissue clearance shifts flow towards the internal pathway. Tail loop and side‐hole diameter modifications exhibited negligible impact on total flow (± <6%), indicating that these features can be modified for better anchoring without significantly influencing drainage performance (Figure 2G,H).

#### 2.1.2 Parametric Study of Abscess Sizes and Symmetry

We varied circular abscess diameters from 40 to 300 mm and oval abscess aspect ratios from 0.5 to 3. Despite a 750% increase in abscess diameter, the change in total fluid flow was less than 2% (Figure 2I). Similarly, varying aspect ratios from 0.5 to 3 resulted in a total fluid flow change of less than 3% (Figure 2J). These data suggest that the instantaneous drainage flow rate is not affected by the specific shape and size of the abscess cavity under constant pressure. This result is expected under our pressure‐driven inlet condition with static walls, where the abscess cavity acts as a reservoir that maintains pressure and does not restrict flow. However, the total abscess volume remains a critical determinant of the total drainage duration.

### 2.2 Fabrication and Manufacturing

**FIGURE 3:**
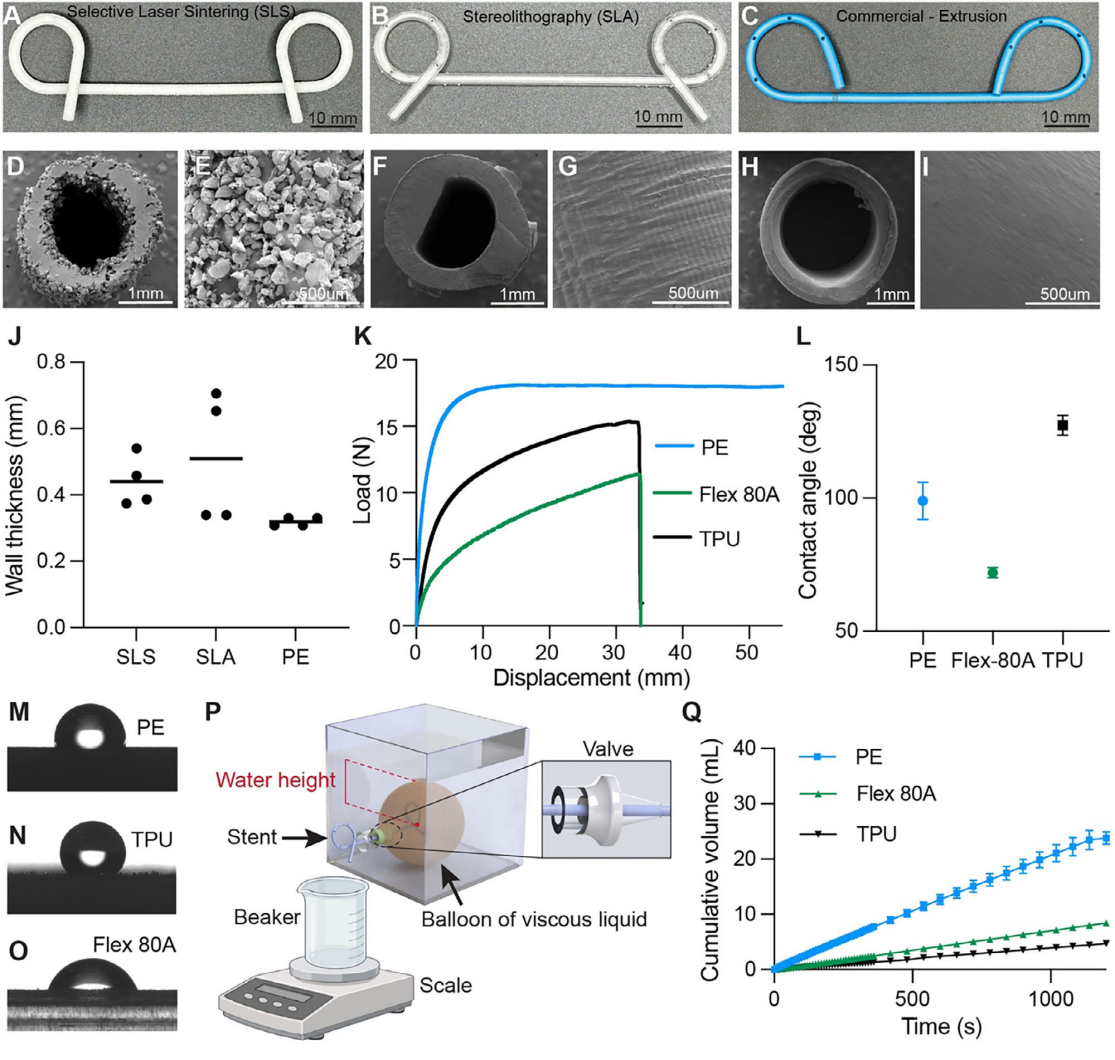
Material characterization and performance criteria. Prototype DPS fabricated via (A) selective laser sintering (SLS) with polyurethane (TPU) and (B) stereolithography (SLA) with Flexible 80A resin. (C) Boston Scientific 7Fr Advanix Biliary Stent (control). (D–I) Scanning electron microscopy (SEM) cross‐sections of (D,E) SLS‐TPU, (F,G) SLA‐Flexible 80A, and (H,I) commercial stent surface microstructures. (J) Wall thickness measurement. (K) Samples of uniaxial tensile testing load‐displacement curve (10 mm/min). (L) Quantitative contact angles measured (n = 3). (M–O) Static contact angle measurements using 5 µL distilled water droplets, (M) PE (N) SLS‐TPU (O) SLA‐Flexible 80A. (P) Experimental fluid dynamics testing platform. (Q) Time‐dependent cumulative drained volume comparing commercial PE and 3D‐printed stents (n = 3).

We next sought to develop additive manufacturing (AM) approaches to mimic the dimensions and mechanical characteristics of a clinical‐grade double‐pigtail stent (DPS). Commercial DPSs are typically extruded from polyethylene, polyurethane, polytetrafluoroethylene, or proprietary blends of these polymers by major manufacturers such as Cook Medical, Boston Scientific, and Olympus. In this study, we selected a polyethylene DPS (7 Fr Advanix Biliary Stent, Boston Scientific) as our benchmark, as it represents a widely used device for endoscopic internal drainage in current clinical practice and was readily available for benchtop testing. To develop AM analogues with material properties comparable to commercial devices yet amenable to 3D printing, we fabricated stent specimens from a polyurethane (TPU 1301, EOS GmbH) using selective laser sintering (SLS), and from Flexible 80A (Formlabs), a flexible, SLA‐printable photopolymer whose proprietary formulation consists of 75–95 wt.% acrylate monomers, 3–6 wt.% urethane dimethacrylate, and <1.5 wt.% photoinitiator, using stereolithography (SLA) (Figure 3A,B) [18]. These AM‐fabricated constructs were then compared against the extruded polyethylene DPS, which served as the clinically relevant control benchmark (Figure 3C).

#### 2.2.1 Surface Morphology Assessment

Scanning electron micrograph (SEM) images of SLS‐TPU showed a non‐uniform wall thickness, high surface roughness, and heterogeneous porous microstructure characteristic of the powder bed fusion process (Figure 3D,E). The central sintered track can be seen as a densely fused continuous track with minimal porosity. Outer edges are lined with partially sintered loose particles and increased porosity. The SLA‐Flexible 80A showed minimal surface roughness with visible layer lines (Figure 3F,G). The Extruded‐PE sample images showed the smoothest surface finish out of all samples with uniform edges (Figure 3H,I). We also measured the wall thickness distribution of each sample based on the cross‐sectional images (Figure 3J). The designed wall thickness is 0.32 mm, equivalent to commercial Extruded‐PE DPS. SLS‐TPU and SLA‐Flexible 80A had average wall thicknesses 37.7% and 59.5% larger than designed, respectively. Therefore, 3D‐printed stents possess more occluded internal lumens than the Extruded‐PE stent.

#### 2.2.2 Mechanical Properties Assessment

We evaluated the mechanical performance of the printed specimens by measuring their elastic modulus and ultimate tensile load (Figure 3K). The Extruded‐PE sample exhibits a short linear elastic deformation followed by a long, near‐constant load plastic deformation plateau, suggesting highly ductile characteristics. The mechanical behavior of the SLS‐TPU sample shows a short linear elastic deformation region, less than 1 mm (5% strain), with a low elastic modulus of 35.43 ± 5.56 MPa, which captures the material's flexibility and porous microstructure from incomplete particle fusion during the sintering process. The SLA‐Flexible 80A sample demonstrates viscoelastic behaviors characterized by a brief linear viscoelastic deformation region followed by a non‐linear viscoelastic time‐dependent transition. Table 1 summarizes the mechanical properties of all tested materials.

**TABLE 1:** Summary of tensile testing results.

Manufacturing method – Material	Elastic modulus (MPa) Assessed at 5% strain	Ultimate load (N)	Area (mm^3^)	Tensile Strength (MPa)
Extruded‐PE (Commercial)	88.31 ± 8.35	19.49 ± 2.20	1.94	10.06 ± 1.13
SLS‐TPU	35.43 ± 5.56	15.99 ± 2.99	2.48	6.46 ± 1.21
SLA‐Flexible 80A	13.31 ± 0.61	11.91 ± 2.68	2.72	4.38 ± 0.99

#### 2.2.3 Contact Angle Measurement

To understand the surface's interaction with fluids and its impact on drainage efficacy, we assessed the surface wettability of printed specimens by measuring the static contact angle of a distilled water droplet on each surface. The SLA‐Flexible 80A sample yielded the smallest contact angle (72.0° ± 1.9°) and the flattest droplet shape, indicating the most hydrophilic surface, followed by Extruded‐PE (99.0° ± 7.0°), and SLS‐TPU is the most hydrophobic (127.4° ± 3.7°) and the roundest droplet shape (Figure 3L–O).

#### 2.2.4 Fluid Evacuation Performance of Commercial and Additively Manufactured Standard DPS

We next assessed the stents’ fluid‐draining efficacy by measuring the cumulative weight of abscess fluid phantom drained from the benchtop GL model over time (Figure 3P). The flow rates of the Extruded‐PE, SLA‐Flexible 80A, and SLS‐TPU stents were 20.8 ± 0.1, 7.0 ± 0.02, and 3.9 ± 0.04 µL s^−1^, respectively (Figure 3Q). SLA‐Flexible 80A stent possesses the mechanical property, surface finish, and appropriate printing resolution closest to commercial DPS's functionality.

### 2.3 Optimization Strategy for Device Design

Based on our 2D CFD simulation results, we identified stent length and inner diameter as critical parameters affecting flow rates. We next developed an analytical framework to estimate the systemic hydraulic resistance of each stent design as a function of stent length and cross‐sectional features. We explored various stent designs while keeping several key criteria and constraints in mind to ensure clinical relevance and manufacturability. To ensure technological adoption and minimize the surgeon's learning curve, we designed the device to maintain compatibility with current clinical endoscopic standards. Consequently, the design incorporates a main lumen exceeding 1 mm in diameter to accommodate guidewire placement. Furthermore, to facilitate a fair comparison across all design iterations, every stent design maintains a consistent outermost diameter of 2.3 mm, equivalent to a 7 Fr stent size.

#### 2.3.1 Analytical Framework

**FIGURE 4:**
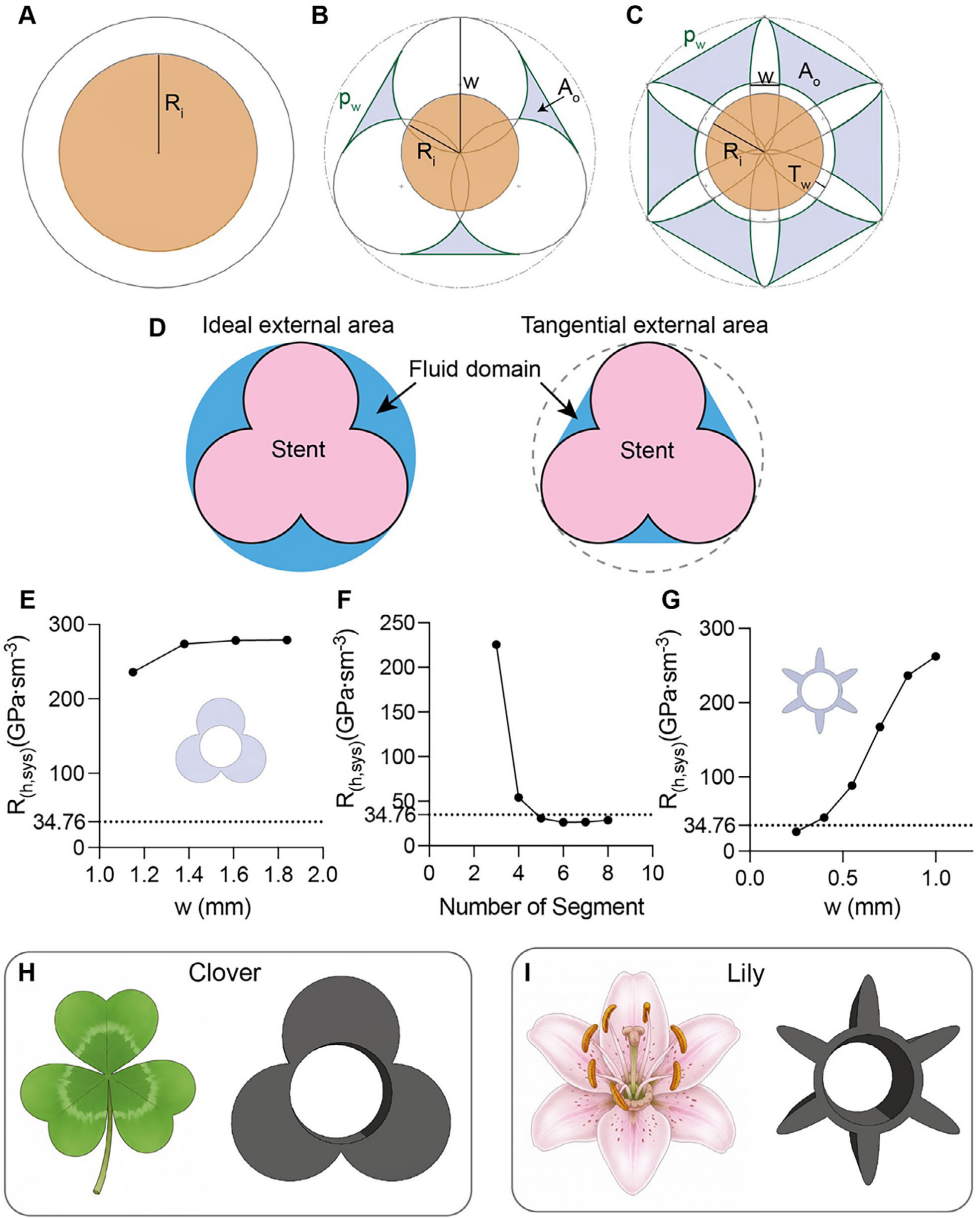
Stent redesign analysis. (A–C) Cross‐sectional schematics: (A) Round, (B) 3‐segment PETALS, and (C) 6‐segment PETALS configurations. (D) Comparison of ideal external area (circular, 2.3 mm diameter) and tangential external area geometry, where cross‐sectional segments connect tangentially at section junctions—accounting for biological tissue's flexible wrapping behavior. (E,F) Analytical comparison of equivalent hydraulic resistance for (E) 3‐segment PETALS, (F) 3–8 segment PETALS of w = 0.25, and (G) 6‐segment PETALS designs across dimensional parameters. (E–G) The dashed line (y = 34.76) represents the hydraulic resistance of a reference commercial stent. (H,I) Floral inspiration and their corresponding shortlisted cross‐section designs, designated “Clover” *N3_W1.15_E1* and “Lily” *N6_W0.25_E0.22*.

To estimate the performance of a drain design, we quantified the hydraulic resistance of the system using a modified form of Hagen‐Poiseuille's law that accounts for both internal and external flows through non‐circular pipes. Refer to Figure 4A–C for the notation. Assuming steady, viscous, and laminar flow of a Newtonian fluid, we begin with the fundamental definition of hydraulic resistance *R_h_*, which is a function of pressure drop Δ*P* and flow rate *Q*.

(1) $$R_{h}=\frac{\Delta P}{Q}$$

This is followed by the definition of Reynolds number, where ρ is fluid density, *V* is fluid velocity, *D_h_* is hydraulic diameter, and µ is the fluid's dynamic viscosity.

(2) $$Re=\frac{\rho VD_{h}}{\mu }\;$$

The Darcy‐Weisbach equation describes the pressure drop due to frictional losses in a pipe, where *f* is the friction factor. Where *L* is the pipe length.

(3) $$▵P=f\frac{L}{D_{h}}\frac{\rho V^{2}}{2}$$

The friction factor for laminar flow in a circular pipe is a function of the Reynolds number.

(4) $$f=\frac{64}{Re}\;$$

The flow rate is related to the velocity and cross‐sectional area *A*.

(5) Q=V·A

Combining Equations (1) through (5), the flow through a non‐circular pipe can be modeled by a modified Hagen‐Poiseuille equation:

(6) $$Q=\frac{A^{2}D_{h}}{32\mu L}\cdot \Delta P$$

The hydraulic diameter of a non‐circular pipe is given by the following expression, where *p* is the wetted perimeter.

(7) $$D_{h}=\frac{4A}{p}\;$$

The hydraulic resistance of internal flow is equivalent to that of a pipe with circular cross‐sectional area.

(8) $$R_{h,in}=\frac{128\mu L_{i}}{\pi {D_{i}}^{4}}\;$$

The hydraulic resistance of external flow is equivalent to that of a pipe with a non‐circular cross‐sectional area.

(9) $$R_{h,out}=\frac{32\mu L_{o}}{A^{2}{D_{h}}^{2}}=\frac{2\mu L_{0}p^{2}}{A^{4}}\;$$

To calculate the equivalent hydraulic resistance of a system with a parallel pipe,

(10) $$\frac{1}{R_{h,sys}}=\frac{1}{R_{h,out}}+\frac{1}{R_{h,in}}$$

The final simplified form of systemic hydraulic resistance is as follows.

(11) $$R_{h,sys}=\frac{128\mu L_{i}L_{o}p^{2}}{\pi L_{o}{D_{i}}^{4}p^{2}+64A^{3}L_{i}}\;$$

To estimate Reynolds numbers for both circular and non‐circular conduits, we used the following formulation, obtained by combining Equations (2), (5), and (7).

(12) $$Re=\frac{4\rho Q}{p\mu }\;$$

We employed this analytical approach to estimate the hydraulic resistance of multiple stent designs. We developed the PETALS (Personalized Endoscopic Transmural Abscess Leak Solution) framework to model stent geometry defined by the number of segments (*n*), characteristic width (*w*) in mm, and eccentricity (*e*). To reference the specific geometric configurations explored in this study, we implemented a parameter‐based nomenclature as follows *N(n)_W(w)_E(e)*. The tangential area *A_o_* was defined as the area enclosed by straight lines tangent to the points of contact between the stent and the 2.3 mm diameter outer boundary (Figure 4D) to account for tissue compliance over the stent. For comparison, the hydraulic resistance of a commercial DPS (Figure 4A) using Equation (11) to be 34.76 GPa·s·m^−^^3^, which we plot as a horizontal line on Figure 4E–G.

We first considered a 3‐segment circular design (*e* = 1), varying w from 1.15 to 1.84 mm (Figure 4E). Our result revealed that hydraulic resistance slightly increased from 236.08 to 273.90 GPa·s·m^−^^3^ as *w* increased from 1.15 to 1.38 mm, then plateaued near 279 GPa·s·m^−^^3^. The minimum *R*_*h*,*sys*_ was found at the lower design limit *w* = 1.15 mm for *N3_W1.15_E1* design. Recognizing the strong dependence of the tangential area on the number of segments, we analyzed the hydraulic resistance of the design with *n* = 3 to *n* = 8, while holding *w* = 0.25 mm and *e* = 0.22 (Figure 4F). A significant drop in *R*_*h*,*sys*_ was observed when *n* increased from 3 to 4, with 6 segments achieving the lowest resistance. Consequently, we explored a 6‐segment ellipse design by varying w from 0.25 to 1.0 mm, corresponding to an *e* range of 0.22 to 0.87, showing that *R*_*h*,*sys*_ increased as *w* increased (Figure 4G). Hence, the minimum *R*_*h*,*sys*_ for this configuration occur at its lower design limit *N6_W0.25_E0.22*. The shortlisted samples *N3_W1.15_E1* and *N6_W0.25_E0.22* were named “Clover” and “Lily”, respectively, based on their morphological resemblance to the corresponding flora (Figure 4H,I).

#### 2.3.2 Computational Analysis of Fluid Drainage Efficacy in Redesigned Stents

**FIGURE 5:**
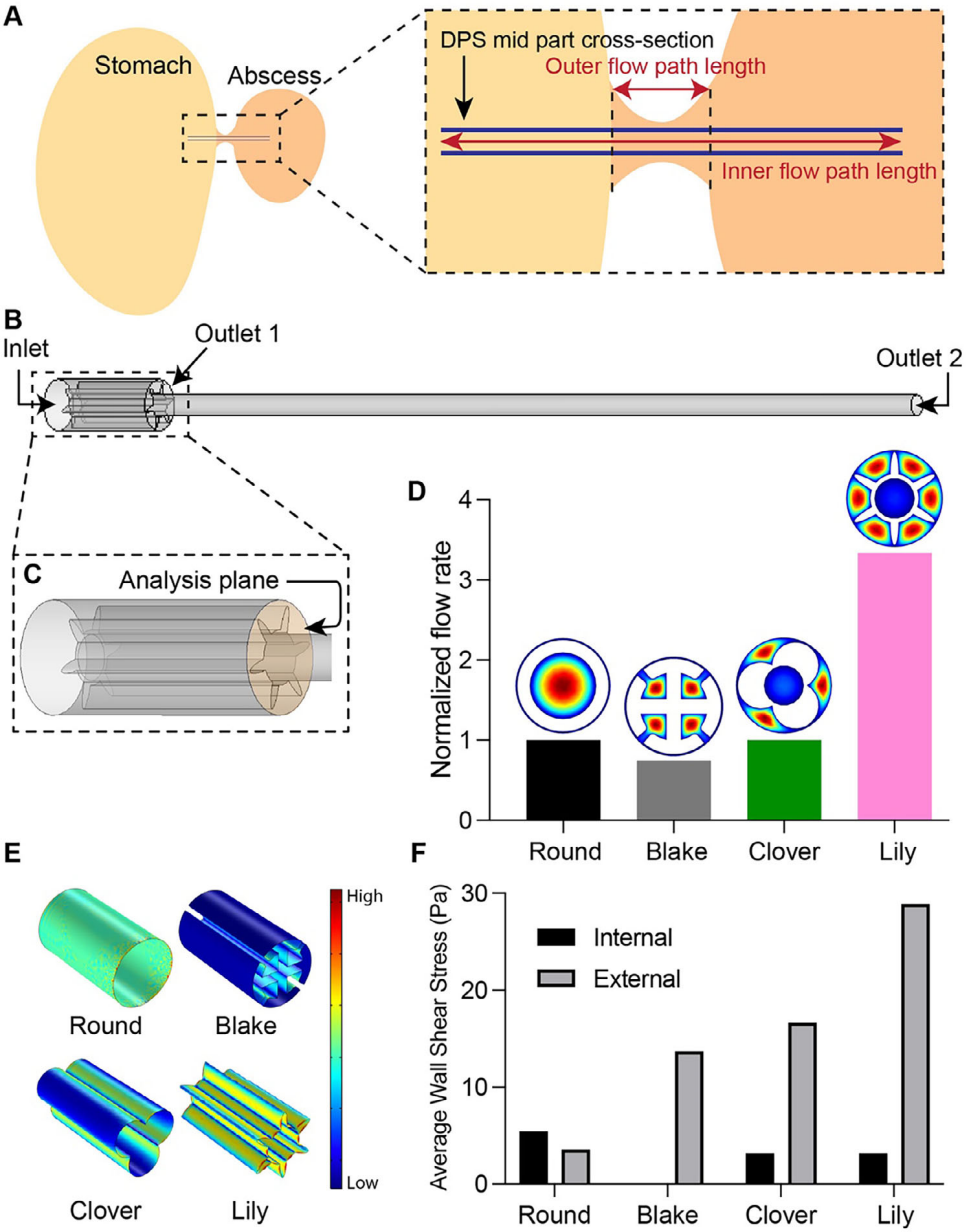
3D CFD simulation framework. (A) Simulation setup focusing on the abscess‐stomach connection and stent position. Zoomed‐in schematic highlights critical flow path lengths (outer and inner flow), which are GL anatomy dependent. (B) Simplified fluid domain geometry with 4 mm outer flow path and 50 mm inner flow path. Boundary conditions include a combined inlet and two outlets, separating external and internal flow. Flow rate was calculated using surface integration of the velocity at the analysis point (C). (D) Volumetric flow rate comparison of commercial round DPS, commercial Blake drain, Flexible 80A – Clover, and Flexible 80A – Lily stents. (E) COMSOL surface plot of wall shear stress distribution. (F) Average wall shear stress at the external and internal walls of all stents.

We developed a simplified model of the gastric leakage for 3D simulation, focusing on the interaction between the stomach and an abscess cavity with an installed stent. This model prioritized critical parameters, including the inner and outer flow path lengths and the cross‐sectional area of the tissue opening. We presented this simplified system as two concentric pipes of unequal length to accurately reflect the significant difference between the internal and external flow path lengths (Figure 5A,B). We quantified the volumetric flow rate for each design configuration by calculating a surface integration of the velocity profile across a defined analysis plane (Figure 5C).

The concept of medical drains incorporating open channels is well‐established. For a comprehensive performance benchmark, we also simulated a proximal end of a Blake drain, a common closed‐suction surgical drain with four continuous external channels along a solid silicone core. Blake drains inherently have no internal hole for guidewire placement. The simulation results demonstrate that our Lily design achieved a flow rate 3.3 times greater than that of the commercial round stent (Figure 5D). Therefore, we identified the Lily design as the top performer, offering substantially higher flow rates than both the commercial round stent and the Blake drain.

In addition to evaluating volumetric flow rate, we also derived the average wall shear stress on both the external and internal walls of each stent design. For a Newtonian liquid, wall shear stress is a function of viscosity and flow velocity gradient at the wall (Equation 13).

(13) $$\tau_{w}=\mu \left(\frac{du}{dy}\right)\;$$

Figure 5E,F illustrate the surface plots of wall shear stress distribution and the corresponding average values for each geometry. We observed that the average wall shear stress of the Lily prototype is 28.89 Pa for the external wall and 3.17 Pa for the internal wall.

### 2.4 Fabrication and Performance Assessment of Redesigned Stents

**FIGURE 6:**
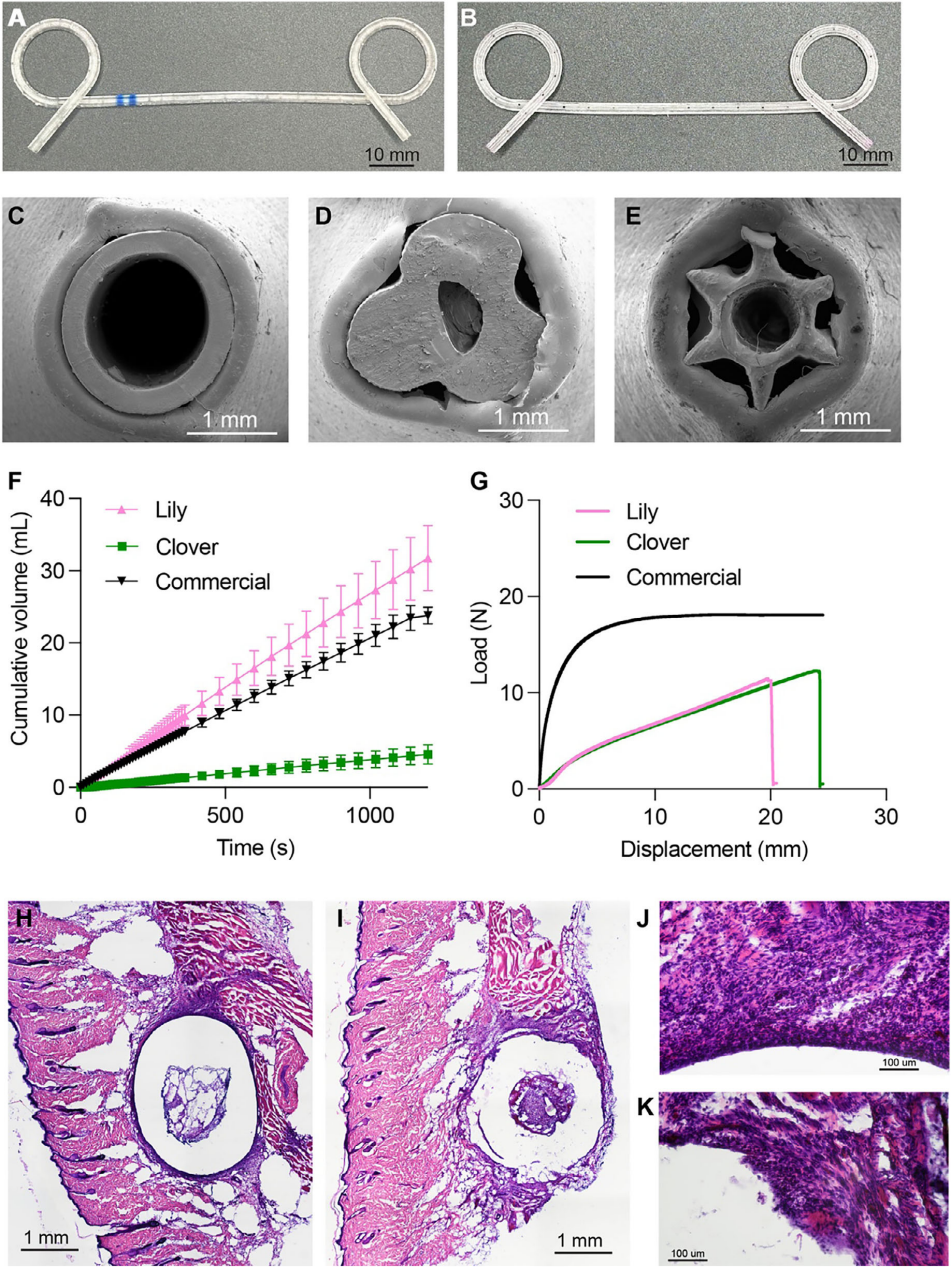
(A,B) 3D printed (SLA) stents of 2 final candidates, (A) Clover and (B) Lily. (C–E) Post‐deployment SEM evaluation of stent‐valve interface patency. (F) Comparative drainage efficacy showing Lily/Clover prototypes achieving 134%/19% flow rate versus commercial DPS. (G) Samples of uniaxial tensile testing load‐displacement curve (10 mm/min). Hematoxylin and eosin–stained skin tissue sections at implantation sites: 5× magnification of (H) commercial polyethylene (PE) control and (I) Flexible 80A, and 20× magnification of (J) commercial PE control and (K) Flexible 80A.

We fabricated the shortlisted Clover and Lily designs using SLA with Flexible 80A resin (Figure 6A,B). The Lily design featured fenestrations, positioned at 5 mm intervals along its length, to preserve a clear central lumen for guidewire placement during the fabrication process. To investigate the mechanical interaction between each stent and the surrounding valve tissue, we inserted the straight portion of each stent into a separate valve and captured top‐view SEM images of the cross‐section (Figure 6C–E). The SEM images revealed that the commercial round DPS maintained high patency, concentrated within its central lumen. In contrast, the Clover design showed minimal opening in both the central and surrounding regions. Finally, the Lily design showed an opening in both the central and surrounding areas, where the tip of each segment touches the valve. This observation supports the validity of using tangential area to capture effective flow pathways with compliant tissue wrapping around the stent, rather than idealized external area, in our analytical approach.

In our subsequent benchtop fluid flow test, the Lily design achieved a flow rate 1.3 times greater than that of the commercial stent (commercial DPS (20.8 ± 0.1 µL·s^−^^1^), Lily (27.0 ± 0.2 µL·s^−^^1^), and Clover (3.80 ± 0.07µL·s^−^^1^)) (Figure 6F). These quantitative flow measurements are consistent with the effective luminal opening observed in the SEM images. Using Equation (11), we calculated the expected flow rate for the commercial stent (23.0 µL·s^−^^1^), Lily (30.4 µL·s^−^^1^), and Clover (3.39 µL·s^−^^1^). The theoretical values differed from the mean experimental measurements by 11–13%. The discrepancies between CFD results, analytical results, and experimental results can be attributed to flexible valve mimicking tissue compliance, printing tolerances, and surface microfeatures. Furthermore, both the Lily and Clover designs demonstrated suitability for clinical handling, with ultimate loads of 11.19 ± 0.6 N and 11.08 ± 1.3 N, respectively (Figure 6G). These values are comparable to the handling forces required during endoscopic deployment and are sufficient to prevent stent damage during clinical use.

### 2.5 Material Biocompatibility: Short‐Term In Vivo Implantation

We conducted an in vivo experiment to assess the local tissue response and biocompatibility of subcutaneously implanted commercially polyethylene tubes and 3D‐printed Flexible 80A tubes after 7 days. Hematoxylin and eosin–stained sections of the dorsal skin at the implantation sites show preserved overall skin architecture for both the commercial polyethylene (PE) and Flexible 80A tubes. In both groups, a discrete implant tract was evident, surrounded by a thin fibrous capsule and an accumulation of cells. The epidermis remained intact without ulceration or visible pus pocket, and no abscesses or extensive tissue necrosis were observed in either condition (Figure 6H–K).

## 3 Discussion

Despite the widespread use of off‐label double‐pigtail stent (DPS) for post‐bariatric gastric leaks, no dedicated endoscopic internal drainage (EID) device has yet been developed for this application. This study addresses this unmet clinical need using additive manufacturing (AM) to create a customizable DPS. These optimized, patient‐specific stents are based on gastric leak (GL) anatomical parameters (connective pathway length and diameter) and developed using the PETALS (Personalized Endoscopic Transmural Abscess Leak Solution) framework, a methodology combining Computational Fluid Dynamics (CFD) and analytical methods. Our redesigned stent, Lily, features a flower‐like cross‐sectional structure that reduces hydraulic resistance, achieving a 30% increase in fluid evacuation efficiency compared to traditional DPS (Figure 6F). Although clinical abscess drainage outcomes are also influenced by factors such as deployment technique, migration tendency, and post‐placement management, these conditions were beyond the scope of our work. Here, we focus on integrating computational and experimental validation to demonstrate that non‐tubular, 3D‐printed stents can enhance drainage performance while preserving procedural compatibility.

Our CFD analysis revealed that stent length and internal diameter are the primary geometric determinants of drainage performance, while tail loop geometry has minimal hydraulic impact but remains critical for anatomical anchoring (Figure 2C–J). This finding strategically decouples the optimization of drainage efficacy from anchoring design, enabling independent refinement of each function. This is essential because endoscopic internal drainage with DPS carries a 13.8% adverse event rate, mostly migration‐related, with rare incidents of perforation and bleeding [9]. Accordingly, future work will focus on anti‐migration geometric optimization. This approach fundamentally reengineers stent morphology, diverging from procedural tactics like using larger or multiple stents [19]. AM enables a broader stent ‘design space’ that is challenging to access with extrusion‐based manufacturing. For example, stents could incorporate spatially varying geometry along their length, such as a graded stiffness, to balance flexibility, kink resistance, and drainage performance. It also permits rapid fabrication of patient‐specific anchoring tail geometries that could be tailored to irregular leak tracts or cavities. These concepts can be further guided by computational shape optimization and finite element analysis (FEA) informed by patient computed tomography to improve conformal fit and stability in anatomically complex cases.

Our 3D‐printed stents also offer a lower elastic modulus than commercial polyethylene stents. Clinical reports indicate a preference for ureteral over biliary double‐pigtail stents (DPS) in certain cases due to their superior flexibility, which reduces tissue and vascular damage [9]. However, ureteral double‐pigtail stents are generally longer (minimum of 10 cm) than biliary stents to accommodate the length of the ureter. According to our 2D CFD results, this increased length can compromise fluid evacuation performance (Figure 2C). With an AM approach using flexible material, we can achieve superior flexibility without sacrificing drainage efficiency. The material candidates, EOS TPU 1301 and Formlabs Flexible 80A, were selected for their elastomeric behavior, which allowed us to evaluate the mechanical performance and printability of the stent design. EOS TPU 1301 has passed cytotoxicity (neutral red assay – ISO 10993–5:2009), in vitro skin irritation (human skin model – OECD 439), and sensitization testing (local lymph node assay – ISO 10993‐10:2013 and OECD 429), according to the manufacturer's declaration of compliance regarding biocompatibility tests [20]. Formlabs Flexible 80A is an engineering‐grade material commonly used for functional prototyping rather than clinical use. Formlabs offers a closely related material with very similar nominal mechanical properties, BioMed Flex 80A, a medical‐grade resin formulation manufactured in an FDA‐registered, ISO 13485–certified facility and tested to ISO 10993 and USP Class VI standards for biocompatibility. This material is declared suitable for long‐term skin contact (> 30 days) and short‐term mucosal membrane contact (< 24 h), indicating a potential pathway to biocompatibility for future iterations [21]. Additionally, our acute implantation of Flexible 80A and commercial polyethylene tubes shows no significant differences in tissue histology after 7 days.

The potential clinical impact of improved drainage efficiency is significant. Current protocols require stent replacement every 4–6 weeks until recovery, with patients often undergoing multiple endoscopic sessions [13]. One cohort study of fifty patients reported an average of 3.14 endoscopic procedures over 57.5 days [22]. Frequent drain replacement is necessary to prevent clogging, irritation, bleeding, perforation, and localized or systemic infection. Additionally, extended use of the drain can provoke foreign body reaction and tissue ingrowth, which can cause complications during removal [23]. If our 30% faster drainage rate proportionally reduces healing time to under 40 days, we could eliminate at least one replacement procedure per patient, minimizing anesthesia risks and shortening hospitalization time. Based on a U.S.‐based billing guide, each endoscopic procedure costs approximately $2000–4000, while extended hospitalization adds an average of $3000 per day [24, 25]. Eliminating one procedure and reducing hospital stay by even 5–7 days could save $17 000–25 000 per patient. With an estimated 2 500 gastric leak cases annually in the U.S. requiring EID, widespread adoption of optimized drainage stents could yield healthcare savings of $42.5 million annually, in addition to reducing patient morbidity and improving quality of life.

The concept of exterior channel drainage has been previously investigated. A similar “lumenless” drain, the ViaDuct (GI Supply, Camp Hill, PA, USA), was developed for biliary drainage. The stent design eliminates the central lumen, directing flow along the stent's exterior to address occlusion. Despite this innovation, clinical reports identified stent occlusion from bile sludge and biofilm accumulation as its primary limitation, with reported time to occlusion ranging from 21 to 89 days [26, 27, 28]. For gastric leak management, stent occlusion is a rare complication [9]. The indwelling period for stents in this setting is typically shorter (28–42 days) than in biliary applications [29]. Furthermore, the viscous nature of gastric abscess fluid results in a substantially higher average wall shear stress during stent‐facilitated drainage compared to bile. Average wall shear stress values provide insight into fluid‐surface interactions, particularly the likelihood of biofilm attachment, which can lead to stent occlusion. Studies demonstrate that biofilm growth rate is inversely proportional to wall shear stress, and that a threshold exists above which shear‐induced detachment predominates, leading to a net reduction in adhering bacteria [30, 31]. While critical shear stress values vary by bacterial strain, the threshold is reported to be below 5.09 Pa [32]. For our Lily stent design, the average wall shear stress at the external wall is 28 Pa, significantly exceeding the reported threshold (Figure 5F). Therefore, we do not anticipate occlusion to be a significant issue in this application. In contrast, in a bile environment, our CFD results show average wall shear stress values of 0.026 Pa for the external wall and 0.0029 Pa for the internal wall of the Lily stent. These values fall within the range where biofilm is likely to attach and grow on the stent surface. This suggests that while the Lily design may excel at gastric abscess drainage, its suitability may vary across different physiological environments.

The PETALS framework can be adapted to optimize stents and drains for other indications, such as pancreatic pseudocyst, colonic diverticulitis abscess, and ureteral obstruction [33, 34, 35]. Our results suggest that optimal designs for internal drainage devices may be non‐cylindrical and can be fabricated using AM on a patient‐specific basis. The current state of the art in AM, featuring larger print volumes, higher resolution, and a growing portfolio of biocompatible materials, supports this transition. The exponential growth of the Medical AM market, projected to reach $27.29 billion by 2030 [36], is exemplified by the dental industry's successful integration of patient‐specific 3D‐printed custom aligners and surgical guides [37]. This shift toward AM‐driven precision medicine has lowered costs and validated the regulatory pathway. Leveraging these mature platforms makes creating tailored medical devices, such as the Lily stent, more feasible and efficient, thereby facilitating the clinical adoption of solutions designed using mathematical frameworks like PETALS.

While AM is ideal for customization, the PETALS framework focuses on analyzing stents and drains with a constant cross‐section. This characteristic is strategically advantageous because the derived geometries can be directly integrated into established, high‐throughput conventional manufacturing methods, such as the extrusion processes currently utilized for commercial DPS. This manufacturing compatibility significantly lowers the technical and financial barriers to adoption for existing stent manufacturers, enabling them to leverage validated materials, scalable production volumes, and proven sterilization protocols immediately. Therefore, this approach allows the translation of optimized stent designs into a comprehensive portfolio of off‐the‐shelf sizes and shapes. Providing surgeons with a broader spectrum of these optimized, constant‐cross‐section devices offers more anatomically appropriate options for GL patients without requiring the immediate implementation of complex AM infrastructure.

This study has several notable limitations. Our experimental model focuses on benchtop drainage under controlled boundary conditions and does not capture the full clinical complexity of GL cavities, including dynamic tissue compliance, debris burden, and patient‐to‐patient variability in movements from physical activities. Additionally, while we provide short‐term histologic assessment and leverage manufacturer biocompatibility descriptions, future work will focus on preclinical testing to examine long‐term mucosal‐contact performance and sterilization/aging effects. Preclinical studies could confirm material biocompatibility, a critical factor given the device's long‐term mucosal contact within a dynamic anatomical environment, and large animal GL models can evaluate the predicted performance of designs. Incorporating biodegradable materials in the AM process could also eliminate the need for secondary retrieval procedures. AI‐driven CT segmentation tools could also be used towards an automated scan‐to‐fabrication workflow to engineer GL anatomy‐specific anti‐migration geometries that enhance device stability.

In summary, we address the unmet clinical need for a dedicated EID device for post‐bariatric gastric leaks by introducing the Lily stent, developed through the PETALS framework. The optimized, AM‐fabricated Lily stent achieves a significant 30% increase in drainage efficiency, eliminating at least one endoscopic procedure per patient. This innovation can potentially save substantial annual healthcare expenses, reduce patient morbidity, and shorten recovery times. By validating the Lily stent design using benchtop and computational models, noting that the constant cross‐section geometry suggests compatibility with conventional extrusion, this work establishes a translatable pathway for delivering anatomically appropriate, highly efficient EID devices to clinical practice.

## 4 Materials and Methods

### 4.1 2D CFD Simulation of Flow Mechanics—Standard DPS

To investigate the flow mechanics of the standard DPS, a two‐dimensional CFD simulation was developed using COMSOL Multiphysics 5.2a. The model geometry represented a top‐down, 2D cross‐section of the experimental apparatus, exclusively simulating the fluid domain. This model was divided into four main parts: an abscess cavity located at the top, a stomach cavity at the bottom, an abscess entrance area connecting the two cavities, and the stent's longitudinal cross‐section. The geometry was constructed in COMSOL to be fully parametric, enabling the abscess radius, opening size, loop radius, fenestration size, inner diameter, and length to be easily manipulated for parametric studies. For quantitative analysis, a data extraction line was defined at the interface between the stomach and the abscess entrance area.

Numerical simulations were conducted using COMSOL Multiphysics' Laminar Two‐Phase Flow, Level Set interface [38]. We used Equation (12) to calculate estimated Reynolds numbers as shown in Table 2, assuming *Q*  =  30µ*Ls* ^−1^;  ρ  =  1230 *kgm* ^−3^;  µ  =  0.137*Pa* · *s*.

**TABLE 2:** Estimated Reynolds numbers of 2D CFD setup at different diameters.

Diameter (mm)	Perimeter (mm)	Re
0.01	0.000031416	34.2938242
0.1	0.000314159	3.429382423
1	0.003141593	0.342938242
10	0.031415927	0.034293824

The laminar flow regime was selected based on an estimated Reynolds number between 0.034 and 34.29 with fluids treated as incompressible and immiscible, and gravitational effects neglected. The Two‐Phase Flow, Level Set interface for lamina flow combines two core physics principles to model the immiscible interface between two incompressible fluids: lamina flow, which describes the fluid motion, and the level set method, which tracks and maintains the interface between the two fluids [39]. Lamina flow is governed by the modified Navier‐Stokes equation for incompressible flow.

(14) $$\rho \frac{\partial u}{\partial t}+\rho \left(u\cdot \nabla \right)u\;=\nabla \cdot \left[-pI+\mu \left(\nabla u+\nabla u^{T}\right)\right]+F_{st}$$

where ρ is the density of the fluid mixture, which varies across the two phases and the interface. ***u*** is the velocity vector. *p* is pressure. ***I*** is an identity tensor. µ is the dynamic viscosity. ∇ ***u*** + ∇ ***u** ^T^* is the shear rate tensor. ***F**_st_* is the surface tension force term. Here, we used the simplified form of the continuity equation for incompressible fluid, which implies no net inflow or outflow of mass from any infinitesimal volume within the fluid.

(15) ∇·u=0

The level set method has the following governing equation.

(16) $$\frac{\partial \phi }{\partial t}+u\cdot \nabla \phi =\gamma \nabla \cdot \left(\varepsilon \nabla \phi -\phi \left(1-\phi \right)\frac{\nabla \phi }{\left|\nabla \phi \right|}\right)$$

where ϕ is the level set function, γ is the reinitialization parameter, which is set to 1 by default, and ε is the interface thickness controlling parameter.

The default setup defines density as a volume average.

(17) $$\rho =\rho_{1}+\left(\rho_{2}-\rho_{1}\right)\phi$$

The dynamic viscosity is defined as a volume average as well.

(18) $$\mu =\mu_{1}+\left(\mu_{2}-\mu_{1}\right)\phi$$

The surface tension force is defined as a function of surface tension σ, Dirac delta function δ, curvature κ, unit normal vector **n,** and the surface gradient operator. ∇_*s*_.

(19) $$F_{st}=\sigma \delta \kappa n+\delta \nabla_{s}\sigma$$

We simulated two liquids: gastric juice assigned water‐like properties (ρ = 1000 kg/m^3^; µ = 0.001 Pa·s) based on a range stated in literature, and abscess content modeled as a 75% corn syrup‐water mixture with experimentally measured properties (ρ = 1230 kg/m^3^; µ = 0.137 Pa·s) [40]. We assigned the abscess content to both the abscess cavity and the abscess entrance region, while the stomach cavity was assigned the fluid properties of gastric juice. Boundary conditions included no‐slip walls, a constant pressure inlet of 668 Pa at the abscess cavity walls, 0.4 ms^−1^ inlet velocity in the stomach cavity, and a 0 Pa pressure outlet.

Prior to simulation, a mesh dependency analysis was conducted. We tested for mesh dependency by parametrically running one standard simulation at different mesh resolutions and analyzing the percent change of the final total mass flow value (Figure S1). The mesh convergence criterion was defined as <6% changes in total mass flow between consecutive mesh refinements. The model was verified using experimental results from the benchtop GL setup. The result showed an over‐prediction discrepancy of 16%. An “extra fine” mesh was ultimately chosen for all geometries to balance result accuracy with computational efficiency. With COMSOL's preset, the study was executed in two steps: first, a Phase Initialization step established a stable initial interface between the two fluids; second, a time‐dependent step solved the coupled Navier‐Stokes and level set convection‐diffusion equations to model the transient fluid interaction. We extracted the data for velocity as a function of location and time along a predefined analysis line and subsequently processed them using a custom MATLAB script to calculate the cumulative mass transferred over a 10‐s period.

### 4.2 Recreating a Standard DPS Using Additive Manufacturing Technologies: Baseline Assessment

#### 4.2.1 Commercial Baseline and Digital Modeling

We selected the Boston Scientific Advanix Biliary Double Pigtail Stent (7F outer diameter, 5 cm length), constructed from polyethylene, as the commercial control for this study. To create a digital benchmark for our designs, we performed manual measurements of the commercial stent using a digital caliper and constructed a corresponding 3D model in SOLIDWORKS 2023.

#### 4.2.2 AM Technologies and Material Candidate Selection

Considering the endoscopic delivery mechanism, which requires the stent's pigtails to uncoil and re‐curl, material flexibility was a primary design constraint. Based on this requirement and the availability of suitable flexible materials, we identified two AM technologies: selective laser sintering (SLS) with polyurethane (TPU) and stereolithography (SLA) with Formlabs Flexible 80A resin. These polymer AM techniques are increasingly used in biomedical prototyping and device development because they enable fabrication of complex geometries at clinically relevant feature scales [41].

#### 4.2.3 SLS Fabrication and Post‐Processing

Specimens were manufactured by SLS using TPU 1301 (EOS GmbH) on an EOS FORMIGA P110 with the “EOS_TPU1301_100_000” parameter as recommended by the manufacturer. Parts were automatically oriented within the EOS P110 build volume, and specimens were printed in both x/y and z orientations. The post‐processing workflow began with part extraction from the unsintered powder cake within a dedicated powder recovery station. Initial cleaning involved manual brushing to remove loose powder, followed by media blasting with ceramic beads to further remove leftover loose powder and improve surface finish. A subsequent ultrasonic cleaning in a soap solution removed any residual particles before the parts were air‐dried [42].

#### 4.2.4 SLA Fabrication and Post‐Processing

Specimens were fabricated using a Formlabs Form 3+ printer with Flexible 80A resin. Prints were performed using the manufacturer's “Flexible 80A” material preset with a layer thickness of 0.05 mm. In terms of fabrication constraints, while the Formlabs Form 3+ SLA printer typically requires a minimum unsupported wall thickness of 200 µm, we set our minimum unsupported wall thickness to be 250 µm for the Flex 80A material due to its inherent pliability and increased susceptibility to deformation during the SLA printing process [43]. The corresponding exposure settings were: perimeter fill exposure 36.0 mJ cm^−^^2^, model fill exposure 30.0 mJcm^−^^2^, supports fill exposure 144.0 mJcm^−^^2^, and top surface exposure 72.0 mJcm^−^^2^. The printer operated at a controlled chamber temperature of 35°C throughout the build. Parts were printed in the x/y orientation. For SLA parts, solvent washing and post‐curing are essential to remove uncured resin and complete polymerization, both of which influence final mechanical properties and biocompatibility of the printed objects [44]. The post‐processing protocol involved washing the printed parts in isopropanol for 15 min using an automated Form Wash station. After automatic washing, we manually flush the main lumen of the printed stent with 100% isopropanol using a 5 mL syringe with a 24‐gauge needle attachment. We then used pressurized air to dry the part and clear out any isopropanol and resin residual that may cause blockage. The parts were then UV‐cured in a Form Cure station at 60°C for 20 min to achieve their final mechanical properties. Finally, the support structures were manually removed using small scissors, and the part was separated from the build platform [45].

### 4.3 Material and Mechanical Characterization

#### 4.3.1 Surface Characterization Using Scanning Electron Microscopy

We performed surface morphology imaging using a ThermoFischer Quanta3D scanning electron microscope. Prior to analysis, samples were prepared via gold sputtering with a plasma sputter coater to increase surface conductivity and prevent surface charging for better image resolution.

#### 4.3.2 Contact Angle Measurement

We assessed surface wettability using a contact angle goniometer. For each measurement, a 5 µL droplet of distilled water was deposited onto the 3D‐printed sample surface. The instrument's software then traced the droplet profile to calculate the static contact angle.

#### 4.3.3 Mechanical Tensile Testing

We conducted uniaxial tensile testing using an Instron 5960 universal testing machine equipped with a 50 N load cell and a tensile test grasper fixture. To ensure a direct comparison with the commercial device, samples were printed as tubes with a 2.3 mm outer diameter and a 1.68 mm inner diameter. We set the gauge length to 20 mm and applied a constant displacement rate of 10 mm/min until sample failure. We processed SEM images of the stent cross‐sections in ImageJ to recalculate the printed cross‐sectional areas, which we then used to convert load to stress for each sample type.

### 4.4 Performance Testing on GL Benchtop Model

To experimentally evaluate the drainage performance of our stent designs, we developed a simplified benchtop model that replicates the key mechanics of endoscopic internal drainage for gastric leakage treatment. We designed the model based on direct input from bariatric surgeons at Cleveland Clinic Abu Dhabi and engineered the system to meet several critical requirements. A water column was applied and maintained a constant homogeneous positive pressure around the abscess cavity. A 3D‐printed one‐way valve prevented the simulated abscess content from draining while under hydrostatic pressure prior to stent insertion. To minimize experimental variability between trials, both the valve and abscess balloon components were designed to be easily interchangeable. The simulated abscess cavity consisted of a 12‐inch transparent latex balloon filled with a viscous fluid, connected via a soft silicone tube (10 mm outer diameter, 6 mm inner diameter) to a custom one‐way duckbill valve. We designed the valve in SOLIDWORKS (Figure S2) and fabricated it using a Stratasys J850 printer, employing Agilus 30 material for the flexible flap and VeroClear for the rigid base. To generate physiological pressure, we submerged the abscess assembly in a water column inside a generic plastic container (18 cm length, 10.9 cm width, and 10 cm height). Using the hydrostatic pressure equation,

(20) P=ρgh

where *P* is hydrostatic pressure, ρ is the liquid density, and *h* is the height of the liquid column, we filled the container to a height of 81.4 mm above the abscess assembly to achieve a pressure of 6 mmHg (800 Pa), consistent with typical adult intra‐abdominal [15]. To simulate the viscous content of an abscess cavity, we consulted three surgeons who selected a mixture of 75% concentrated corn syrup in water by volume as a representative fluid. We characterized this mixture and measured its viscosity as 0.137 Pa·s and density as 1230 kg/m^3^. We filled each abscess balloon with 50 mL of this fluid. Assuming a complete depletion of liquid inside the balloon, the change in water level and the corresponding pressure is less than 0.19 mmHg, which is relatively insignificant. For each trial, we installed the abscess assembly inside the container and filled the container with tap water to the predefined height. To deliver the stent into the abscess cavity, we loaded it onto a guidewire, inserted the guidewire through the soft tube and duckbill valve until the start of the loop tail was inside the cavity, then retracted the guidewire, ensuring the loop tail remained entirely within the balloon. During each 20‐min trial, we continuously recorded the mass of evacuated fluid using a digital scale, while a digital camera captured the process. We recorded data points in 5‐s intervals until 360 s, followed by 1‐min intervals until the end of the experiment.

### 4.5 3D CFD Simulation of Flow Mechanics – Stent with Different Cross‐Sectional Geometries

We developed a simplified three‐dimensional (3D) model representing the gastric leak anatomy to computationally assess the performance of new stent cross‐sectional designs. We focused on the connection between the stomach, an abscess cavity, and the straight mid‐section of a DPS, which we modeled as two concentric pipes of unequal length with a shared inlet and two outlets. For this model, we defined the inner and outer flow path lengths as 50 and 4 mm, respectively. We employed Equation (12) to estimate the Reynolds numbers as follows, assuming *Q*  =  30µ*Ls* ^−1^;  ρ  =  1230 *kgm* ^−3^;  µ  =  0.137*Pa* · *s* (Table 3).

**TABLE 3:** Estimated Reynolds number of different cross‐sectional geometries.

Type	Perimeter (mm)	Re
Round	20.04336113	0.053752076
Blake	11.276496	0.095541404
Clover	6.600741	0.163219896
Lily	10.928868	0.098580408

We established a stationary, single‐phase, incompressible laminar flow simulation using the fluid properties of the abscess content (density ρ = 1230 kg/m^3^; dynamic viscosity µ = 0.137 Pa·s) and applied a no‐slip condition to all walls. We generated a physics‐driven mesh using the physics‐controlled mesh with “Normal” element size preset and conducted a mesh dependency analysis to ensure our results were mesh‐independent (Figure S3). To quantify performance, we calculated the volumetric flow rate for each design configuration by performing a surface integration of the velocity profile across a defined analysis plane. Pressure inlet condition at 668 Pa, consistent with typical adult intrabdominal pressure, and 0 Pa for the outlets were applied [15]. For bile conditions, we assigned a less viscous and less dense fluid property according to literature values (density ρ = 1005 kg/m^3^; dynamic viscosity µ = 0.00105 Pa·s) [46, 47]. The inlet condition is defined as 620 mL/day, which is 7.1759e‐9 m^3^s^−1^ [48].

### 4.6 Material Biocompatibility—Short‐Term In Vivo Study

All animal experiments were performed in accordance with protocols approved by the Institutional Animal Care and Use Committee at New York University Abu Dhabi (Protocol 24‐008). Tubes of 1 cm length with 2.3 mm outer diameter and 1.68 mm inner diameter were 3D printed on a Formlabs Form 3+ printer using Flexible 80A resin and post‐processed as described in Section 4.2. For the commercial control, a new, sealed Boston Scientific Advanix polyethylene stent was opened, and 1 cm long straight tube segments were cut from the mid‐section. All implant objects were cold sterilized by submerging in ethanol and exposing to UV light in a biosafety cabinet for 2 h prior to implantation.

For implantation, a Sprague Dawley rat was anesthetized with isoflurane, and the dorsal area was shaved and disinfected with alternating povidone‐iodine (betadine) and ethanol scrubs. Small incisions were made using surgical scalpels and scissors, and two samples of each type were implanted subcutaneously. After implantation, the incisions were closed with sutures. The animal's health was monitored for 7 days following the procedure, after which it was prepared for perfusion.

For perfusion, the anesthetized animal underwent cardiac perfusion with phosphate‐buffered saline, followed by 4% paraformaldehyde (PFA). The skin containing the implantation sites was then harvested, immersed in PFA for 48 h, and subsequently transferred to 30% sucrose solution until the tissue sank to the bottom of the container. The skin samples were embedded in cryo‐embedding medium (Tissue‐Tek) and frozen at −80°C in preparation for cryosectioning. Sections of 30 µm thickness were obtained at −18°C using a cryostat (Leica CM1950) and mounted on SuperFrost Gold microscope slides. To evaluate tissue morphology after implantation, the sections were stained with hematoxylin and eosin (H&E). The H&E images were taken on an Eclipse Ti2‐E Widefield Fluorescence Microscope (Nikon Instruments Inc.).

## Author Contributions

The study was conceptualized by P.P., K.B.R., J.S.B., and C.A.V. The methodology was developed by P.P. and K.B.R., while the investigation was carried out by P.P., Y.K., S.S., H.T.N., A.D., and B.K. Technical guidance was provided by O.K. and M.A., and clinical guidance by J.S.B., J.P.P., A.Z., C.A.V., J.R., and M.K. The original draft was written by P.P. and K.B.R., and subsequently reviewed and edited by J.S.B., J.P.P., O.K., M.A., A.Z., C.A.V., J.R., and M.K. Supervision was undertaken by K.B.R., J.S.B., and C.A.V., and funding was acquired by K.B.R., J.S.B., and C.A.V.

## Funding

This work was supported by New York University Abu Dhabi and Sandooq Al Watan (Grant #F22‐016).

## Conflicts of Interest

The authors declare no conflicts of interest.

## Supporting information




**Supporting File**: adhm71106‐sup‐0001‐SuppMat.docx.

## Data Availability

All data needed to evaluate the conclusions in the paper are present in the paper and/or the Supplementary Materials.
