# Supplementary material for: Enhanced Endoscopic Internal Drainage of Gastric Abscess Through Additively Manufactured Stents
Source: Adv Healthc Mater. 2026 Apr 2;15(21):e05860. doi: 10.1002/adhm.202505860 (PMC13241474; doi:10.1002/adhm.202505860)
Supplement: Supplementary file 1 — Supporting File: adhm71106‐sup‐0001‐SuppMat.docx. [file ADHM-15-0-s001.docx]

*
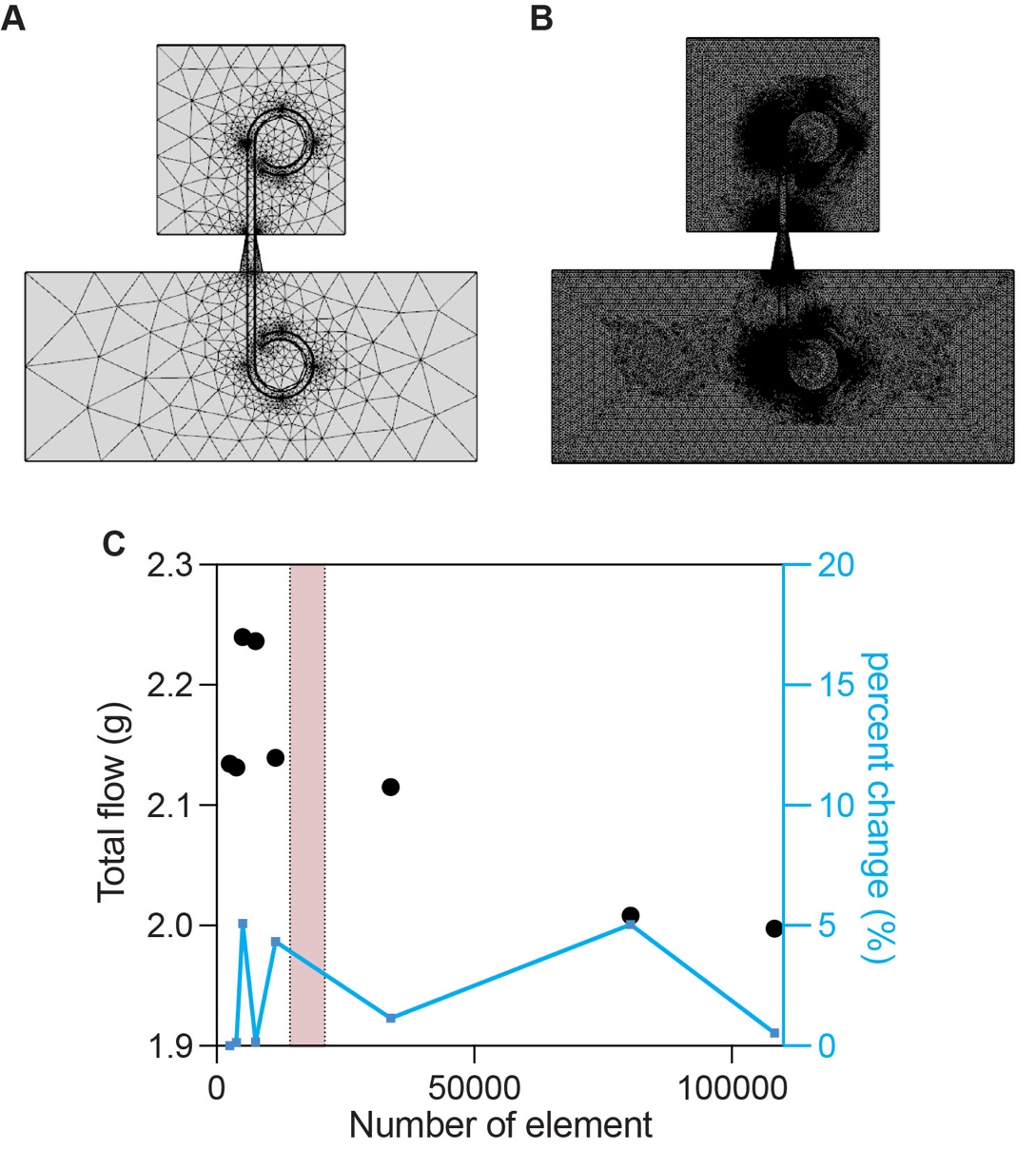
*

***Supplementary Figure 1.*** *Mesh dependency analysis of the 2D CFD setup. (A) “Extremely coarse” mesh. (B) “Extremely fine” mesh. (C) A graph of total flow and percent change at different number of elements.*


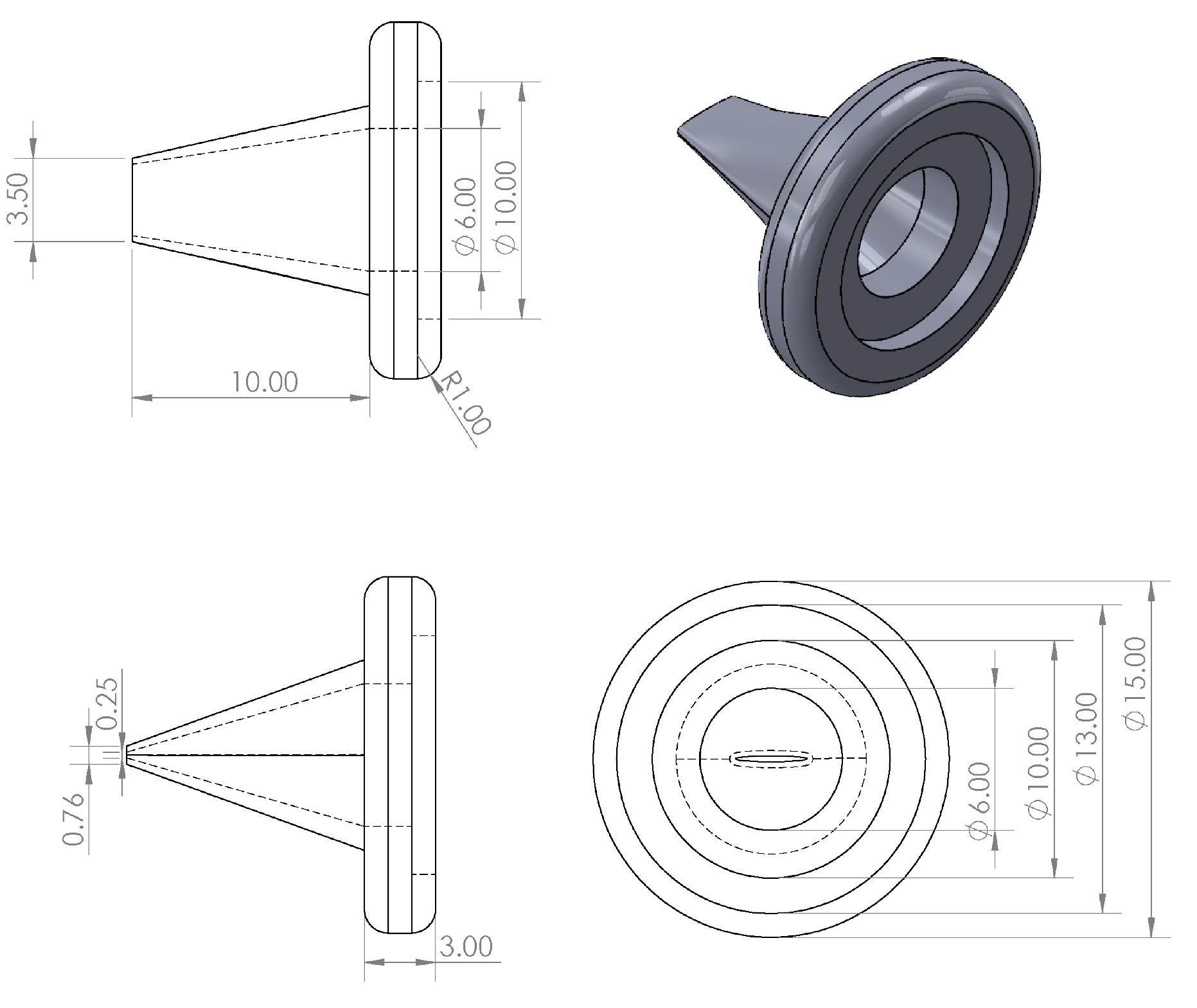


***Supplementary Figure 2.*** *Technical drawing with measurement (mm) of the 3D printed valve on a GL benchtop model.*


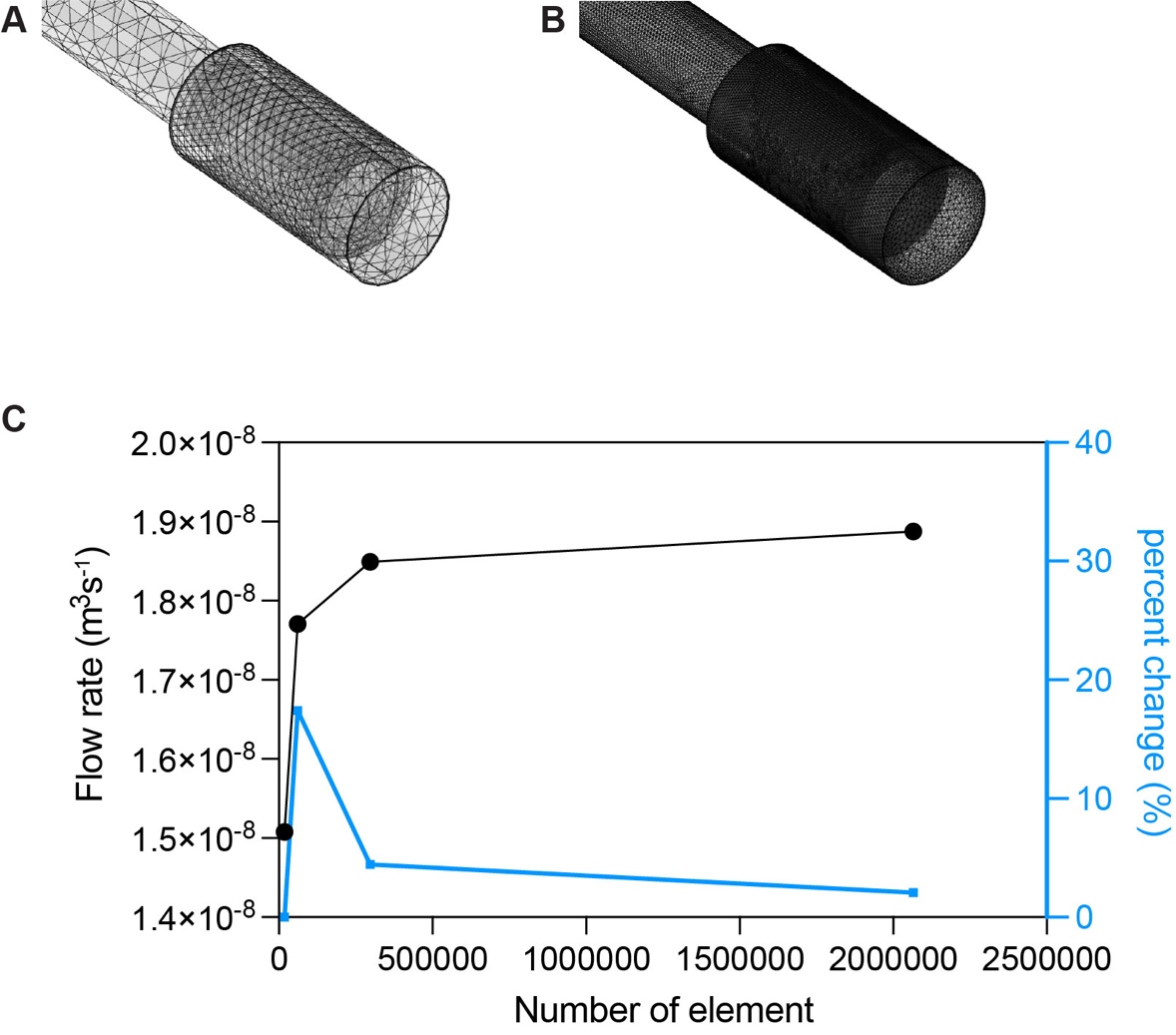


***Supplementary Figure 3.*** *Mesh dependency analysis of the 3D CFD setup. (A) “Extremely coarse” mesh. (B) “Extremely fine” mesh. (C) A graph of total flow and percent change at different number of elements.*

*
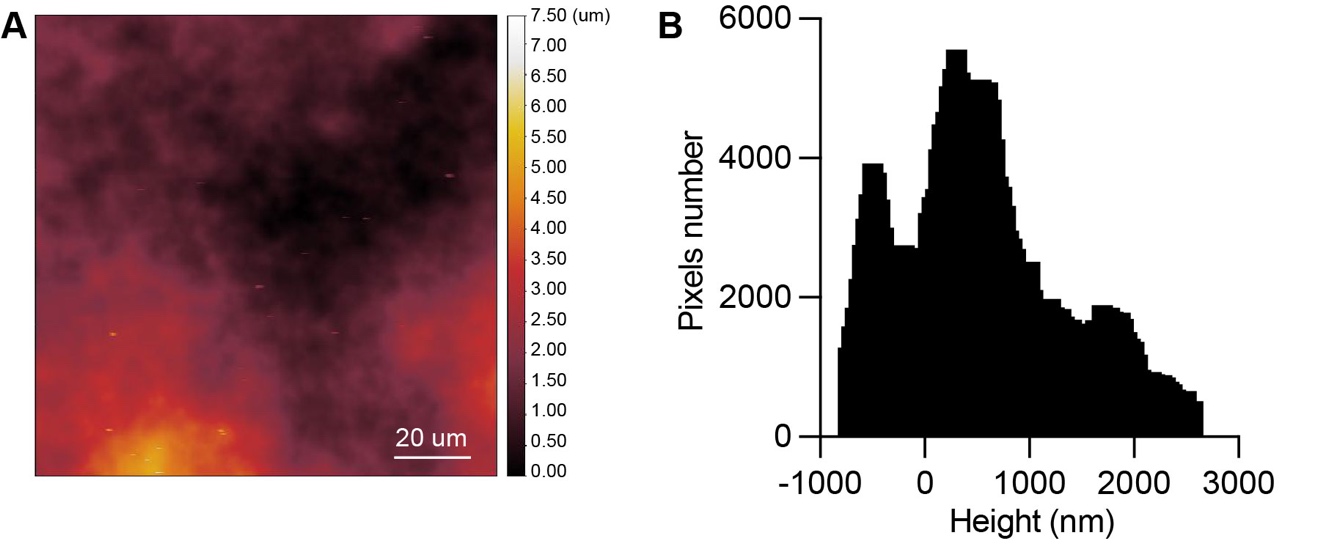
*

***Supplementary Figure 4.*** *Atomic Force Microscopy result. (A) Height map of a SLA-Flex 80A printed square sample. (B) Histogram of height distribution of a SLA-Flex 80A sample. Note that SLS-samples surface roughness was out of range (> 10 μm).*
